# Complete genome sequences of feline astroviruses from asymptomatic stray cats in Japan

**DOI:** 10.1128/mra.00712-24

**Published:** 2024-10-21

**Authors:** Ichika Kitashin, Hitomi Kumano, Keisuke Nakagawa

**Affiliations:** 1 Joint Graduate School of Veterinary Sciences, Gifu University, Yanagido, Gifu, Japan; 2 Laboratory of Veterinary Microbiology, Joint Department of Veterinary Medicine, Gifu University, Yanagido, Gifu, Japan; 3 Education and Research Center for Food Animal Health, Gifu University, Yanagido, Gifu, Japan; 4 Center for One Medicine Innovative Translational Research, Gifu University, Yanagido, Gifu, Japan; Portland State University, Portland, Oregon, USA

**Keywords:** astrovirus, feline, genomes

## Abstract

We determined the complete genome sequences of two feline astroviruses (FAstVs) in non-diarrheic stool samples from apparently healthy stray cats in 2020 in Japan. Information on the complete genome sequence of FAstV from asymptomatic cats has great potential for a better understanding of the ecology of FAstV in cats.

## ANNOUNCEMENT

Astrovirus (AstV) is a non-enveloped virus with a single-stranded positive-sense RNA. AstV belongs to the order family Astroviridae, which comprises two genera, *Mamastrovirus* and *Avastrovirus,* that infect mammals and birds, respectively (1). Feline astrovirus (FAstV), belonging to *Mamastrovirus*, has been detected in feces of cats (*Felis catus*) with or without diarrhea in many countries (2–6). Here, we tried to detect the FAstV genome from 50 non-diarrheic stool samples from apparently healthy stray cats that were provided by the Animal Protection Center in Aichi Prefecture, Japan, in 2020. Consequently, two samples were positive by RT-PCR targeting ORF2 for screening of FAstV (7, 8), and the complete genome sequences of FAstV GU2-27TYT100 and GU2-27TYT101 were successfully determined.

RNAs were extracted from each stool sample using a Direct-zol RNA miniprep kit (Zymo Research). cDNAs were synthesized using SuperScript III reverse transcriptase (Invitrogen) and random primers (Invitrogen). Each of the whole viral genomes, comprising three overlapping amplicons, except for 22 nucleotides (nt) at the 5’ end and 19 nt at the 3’ end, was generated from cDNA using EX Taq HS (TaKaRa Bio) and specific primers. The 5’ end of each of the viral genomes was amplified by using the rapid amplification of cDNA ends (RACE) method (5’-full RACE core set; TaKaRa Bio) following the manufacturer’s protocol. The 3’ end of each of the viral genomes was amplified by using specific primers and anchored oligo(dT) primers. The sequences of all primers used are shown in Table 1. The PCR products were purified using a NucleoSpin Gel and PCR Clean-up Kit (Macherey-Nagel) and were sequenced with a BigDye Terminator v3.1 Cycle Sequencing kit (Applied Biosystems) on an ABI Prism 3100 DNA analyzer (Applied Biosystems).

**TABLE 1:** Primers used in this study

Experiment	Primer name^*a*^	Sequence (5’ to 3’)	Nucleotide position	Cycle condition
RT-PCR (Screening)	Mon269 (Ref. 7, 8)	CAACTCAGGAAACAGGGTGT	4,526–4,545	Denaturation: 94°C, 30 seconds Annealing: 50°C, 30 seconds Extension: 72°C, 60 seconds Number of cycles: 40
	Mon270 (Ref. 7, 8)	TCAGATGCATTGTCATTGGT	4,955–4,974	
RT-PCR (Amplification)	PCR1 F	CCAAAAGAGTGCCTGTGGCTGG	1–22	Denaturation: 94°C, 30 seconds Annealing: 57°C, 30 seconds Extension: 72°C, 60 seconds Number of cycles: 40
	PCR1 R	AGTTCGTTGTATTCCTCCTCGGT	2,063–2,085	
	PCR2 F	CCACAGGGTCTTGAAGCCGA	1,808–1,827	Denaturation: 94°C, 30 seconds Annealing: 57°C, 30 seconds Extension: 72°C, 90 seconds Number of cycles: 40
	PCR2 R	CTTCTTTNCCAGNCTTGCTAGCCAT	4,274–4,298	
	PCR3 F	CAGTTGCTATCCGCTTTTATGG	4,114–4,135	Denaturation: 94°C, 30 seconds Annealing: 42°C, 30 seconds Extension: 72°C, 90 seconds Number of cycles: 40
	PCR3 R	GCTTCTGATTAAATCAATT	6,793–6,811	
3’ RACE	Anchored oligo(dT) RT primer	TCACACCAGCCACGAAAGACA TTTTTTTTTTTTTTTTTTTTTTT	Poly(A)	RT reaction
	F 3’-terminal seq 1 (1^st^ PCR)	CCGCTGAACCTCCTCCTTTTAAGG	6,324–6,347	Denaturation: 94°C, 30 seconds Annealing: 58°C, 30 seconds Extension: 72°C, 30 seconds Number of cycles: 40
	F 3’-terminal seq 2 (2^nd^ PCR)	AAGATCAGCAAGCGTGCCTACCCCA	6,536–6,560	
	R 3’ anker (1st and 2nd PCR)	TCACACCAGCCACGAAAGACA	6,791–6,811	
5’ RACE	5’ phosphorylated RT primer	GATCGCCTCGTACTG	695–709	RT reaction
	5’ RACE S1 (1st PCR)	GGACCATCTTTACTGACATTCG	585–606	Denaturation: 94°C, 30 seconds Annealing: 51°C, 30 seconds Extension: 72°C, 30 seconds Number of cycles: 40
	5’ RACE S2 (2nd PCR)	CTTCGGATTAAGATGGCTCT	653–672	
	5’ RACE R1 (1st and 2nd PCR)	AAGACCAACCCTTGCACCTC	179–198	
Sequencing	M13 F	GTAAAACGACGGGCCAGT		Denaturation: 96°C, 10 seconds Annealing: 50°C, 5 seconds Extension: 60°C, 4 minutes Number of cycles: 25
	FAstV1 F	AGCGACTCTGGCACTTGATAA	409–429	
	FAstV2 F	ATTAAGATGGCTCTCTTTAACGTC	659–682	
	FAstV3 F	GCGCCCGTCCTTGGAGCAGTACTACT	1,010–1,035	
	FAstV4 F	ATGTTGTGACCAACCATAAGA	1,425–1,445	
	FAstV5 F	TAACCGAGGAGGAATACAA	2,061–2,079	
	FAstV6 F	GGAAGTGTGTACCTAACAACTTCCCG	2,831–2,856	
	FAstV7 F	ATGGCTAGCAAGCTGGAAAGAAG	4,274–4,296	
	FAstV8 F	AACTAGCTGGTCCGGGCTTGGAG	4,786–4,808	
	FAstV9 F	GGCATCTGAGTGGTGCTTCACCGGCT	5,023–5,048	
	FAstV10 F	ACCACCCTACAGTTTACACAAATGAA	5,474–5,499	
	FAstV11 F	AAGATCAGCAAGCGTGCCTACCCCA	6,536–6,560	
	FAstV1 R	TCAGTCGACAGGCGGCACACTTC	608–630	
	FAstV2 R	GCGTGGCCTCGGCTCTCAA	1,723–1,741	
	FAstV3 R	CTTCACCTCCTCAATCTTTGG	2,318–2,338	
	FAstV4 R	TCTCCAGATGGAAGGAGGACAT	3,671–3,692	
	FAstV5 R	CTTCTTTNCCAGNCTTGCTAGCCAT	4,274–4,298	
	FAstV6 R	GCTCTCTGACATATTGCTGGTTTAGGTCC	4,502–4,530	
	FAstV7 R	GATAGCTCGTACTCATGCACTAGGTCC	6,247–6,273	

^*a*^ F, forward; R, reverse.

**Fig 1:**
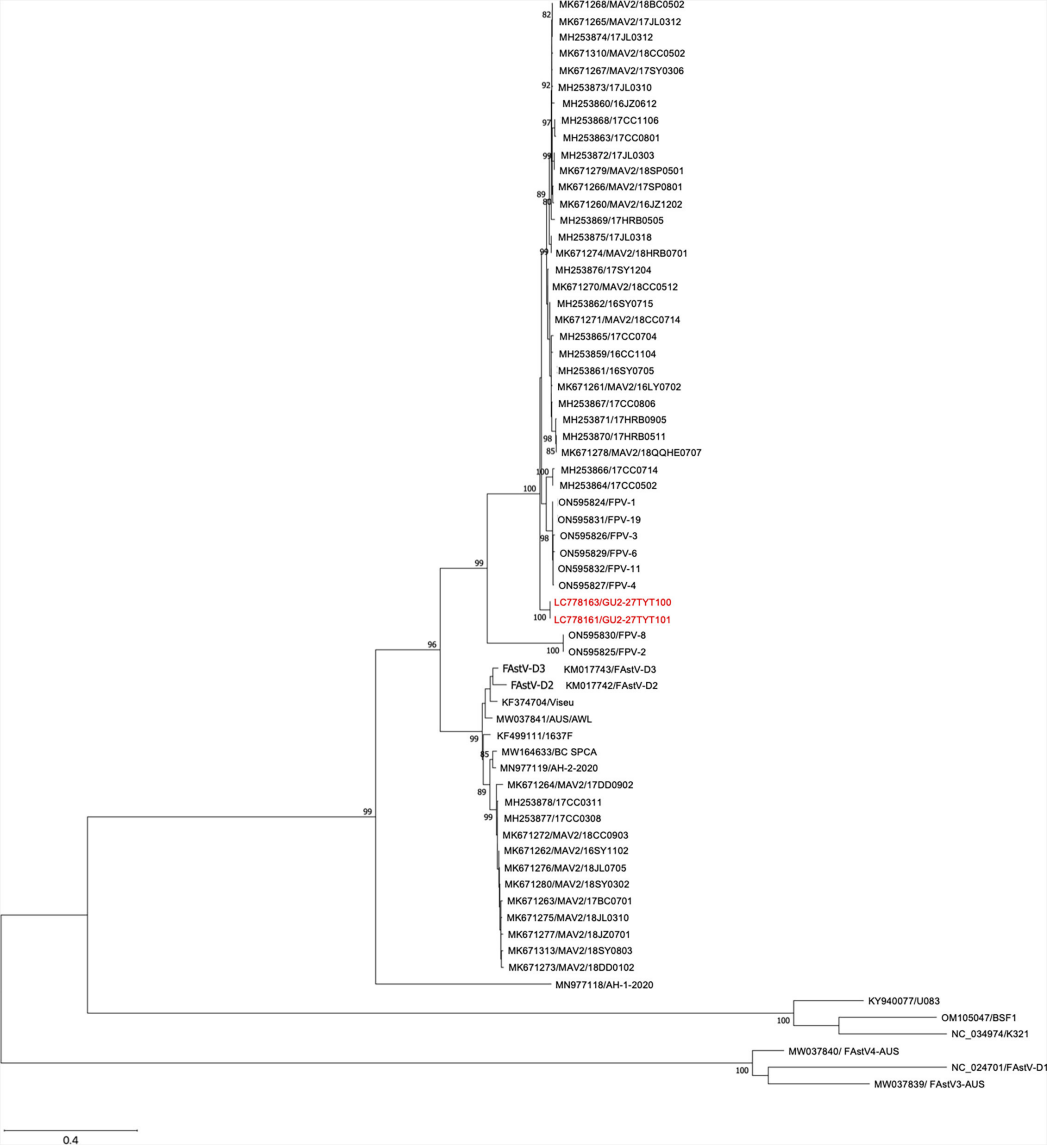
Phylogenetic tree based on amino acid sequences of the entire capsid proteins of FAstVs. The tree was generated by the maximum likelihood method (bootstrap value: 1,000 replicates) using MEGA version XI (9) FAstV GU2-27TYT100 and GU2-27TYT101 are shown in red color.

To cover each of the entire viral genomes, 23–24 Sanger reads were generated, and a quality score of 30 was used as a cutoff value for sequence analysis. The determined sequences were manually assembled using ApE (https://jorgensen.biology.utah.edu/wayned/ape/) with the National Center for Biotechnology Information (NCBI) reference sequence of isolate MAV2/17SP0801 (GenBank accession number MK671309.1), resulting in the following genome organization: 5’ untranslated region (UTR)-ORF1a (nonstructural polyprotein)-ORF1b (RNA-dependent RNA polymerase)-ORF2 (capsid protein) −3’ UTR. The full-length genome sequence of both FAstV GU2-27TYT100 and GU2-27TYT101 comprised 6,832 nt with a G + C content of 49.0%. There were one nt and one amino acid differences in ORF1a between the two FAstVs. A phylogenetic tree based on amino acid sequences of the entire capsid proteins of FAstVs, including FAstVs GU2-27TYT100 and GU2-27TYT101, was generated by the maximum likelihood method using MEGA version XI with default parameters (9) (Fig. 1). FAstVs GU2-27TYT100 and GU2-27TYT101 generate completely separated clusters with a high bootstrap value (100), suggesting that both could be new variants of FAstV.

## Data Availability

The complete genome sequences of FAstVs GU2-27TYT100 and GU2-27TYT101 have been deposited in GenBank under the accession numbers LC778163 and LC778161, respectively. The raw data were deposited under sequence read archive (SRA) numbers DRR590902 and DRR590903, BioSample numbers SAMD00657115 and SAMD00657116), and BioProject PRJDB17005.
